# Hepcidin Plays a Key Role in 6-OHDA Induced Iron Overload and Apoptotic Cell Death in a Cell Culture Model of Parkinson's Disease

**DOI:** 10.1155/2016/8684130

**Published:** 2016-05-19

**Authors:** Qi Xu, Anumantha G. Kanthasamy, Huajun Jin, Manju B. Reddy

**Affiliations:** ^1^Department of Food Science and Human Nutrition, Iowa State University, Ames, IA 50011, USA; ^2^School of Public Health, Shanghai University of Traditional Chinese Medicine, Shanghai 201203, China; ^3^Department of Biomedical Sciences, Iowa State University, Ames, IA 50011, USA

## Abstract

*Background*. Elevated brain iron levels have been implicated in the pathogenesis of Parkinson's disease (PD). However, the precise mechanism underlying abnormal iron accumulation in PD is not clear. Hepcidin, a hormone primarily produced by hepatocytes, acts as a key regulator in both systemic and cellular iron homeostasis.* Objective*. We investigated the role of hepcidin in 6-hydroxydopamine (6-OHDA) induced apoptosis in a cell culture model of PD.* Methods*. We downregulated hepcidin using siRNA interference in N27 dopaminergic neuronal cells and made a comparison with control siRNA transfected cells to investigate the role of hepcidin in 6-OHDA induced neurodegeneration.* Results*. Hepcidin knockdown (32.3%, *P* < 0.0001) upregulated ferroportin 1 expression and significantly (*P* < 0.05) decreased intracellular iron by 25%. Hepcidin knockdown also reduced 6-OHDA induced caspase-3 activity by 42% (*P* < 0.05) and DNA fragmentation by 29% (*P* = 0.086) and increased cell viability by 22% (*P* < 0.05). In addition, hepcidin knockdown significantly attenuated 6-OHDA induced protein carbonyls by 52% (*P* < 0.05) and intracellular iron by 28% (*P* < 0.01), indicating the role of hepcidin in oxidative stress.* Conclusions*. Our results demonstrate that hepcidin knockdown protected N27 cells from 6-OHDA induced apoptosis and that hepcidin plays a major role in reducing cellular iron burden and oxidative damage by possibly regulating cellular iron export mediated by ferroportin 1.

## 1. Introduction

Parkinson's disease (PD) is an incurable neurodegenerative disease that affects more than 1% of people over 65 years old and approximately 4% of the population aged over 80 years [1]. The prevalence is expected to rise sharply within the next two decades because of progressive aging population [2]. Parkinson's disease is characterized by the progressive loss of dopaminergic neurons in the substantia nigra (SN), degeneration of projecting nerve fibers in the striatum, and accumulation of intracytoplasmic inclusions, known as Lewy bodies [3]. Although the etiology of PD is not clear, both genetic and environmental risk factors including exposure to metals and pesticides are considered to be involved in PD [4].

Iron, the most abundant trace metal in the brain, is thought to play an important role in the pathogenesis of PD. Studies have demonstrated the association between iron dysregulation and PD. Increased levels of iron deposits in the SN are observed in postmortem studies [5], as well as in 6-hydroxydopamine (6-OHDA) [6] and 1-methyl-4-phenyl-1,2,3,6-tetrahydropyridine (MPTP) [7] induced PD animal models. The imaging studies of living PD patients also confirmed the presence of accumulation of iron in the SN and linked the extent of iron deposits to the severity of disease [8]. Although iron is important in various physiological functions, such as DNA synthesis, mitochondrial respiration, and oxygen transport [9], free iron is potentially toxic as it is involved in the generation of hydroxyl radicals, which can react with lipid, protein, and DNA, leading to subsequent neuronal damage and death. Moreover, free iron in dopaminergic neurons can accelerate toxic alpha-synuclein fibril formation, leading to neuronal dysfunction [10].

Because of the potential toxicity of iron, iron homeostasis is tightly regulated by a complex system that coordinates iron uptake, release, storage, and utilization. For example, iron is delivered to tissues by circulating transferrin, and excess iron in the cell is stored in the cytosolic ferritin [11]. Hepcidin is a small peptide that is mainly secreted by hepatocytes in response to inflammation, iron overload, and oxidative stress [12, 13]. It controls systemic iron levels by regulating iron absorption from the intestine, the release of iron from degraded hemoglobin from macrophages, and stored iron from hepatocyte [14]. Hepcidin is also considered as a master regulator in the management of cellular iron homeostasis by binding to iron exporter protein ferroportin 1 (Fpn1) in cell membranes and causing its subsequent internalization and lysosomal degradation [3]. Although hepcidin is predominantly expressed in the liver, recent research demonstrates that hepcidin is also widely distributed in the central nervous system. One study showed that hepcidin mRNA level is increased with aging in mouse brain, particularly in the cerebral cortex, hippocampus, and striatum, which leads to decreased level of Fpn1 and the associated iron accumulation in aging brain [15]. Another study showed that peripheral iron overload induces hepcidin and decreased level of Fpn1 in the SN of rats, suggesting the critical role that hepcidin plays in brain iron disturbance [16]. The objective of this study was to determine the role of hepcidin in 6-OHDA induced cell death by knocking down hepcidin expression in N27 dopaminergic cell model of PD.

## 2. Materials and Methods

### 2.1. Chemicals

The immortalized rat mesencephalic dopaminergic neuronal cell line (1RB3AN27, generally referred to as N27) was a kind gift from Dr. Kedar N. Prasad, University of Colorado Health Sciences Center (Denver, CO). RPMI-1640 medium, fetal bovine serum, L-glutamine, penicillin, and streptomycin were obtained from Invitrogen (Carlsbad, CA). Calcein-AM, ascorbic acid, mouse *β*-actin antibody, 6-OHDA, ferrous sulfate, and ascorbic acid were purchased from Sigma-Aldrich (St. Louis, MO). The Amaxa Nucleofector kit was purchased from Lonza (Allendale, NJ). The Absolutely RNA Miniprep kit and High Capacity cDNA Archive kit were purchased from Stratagene (La Jolla, CA) and Life Technologies (Grand Island, NY), respectively. The hepcidin specific siRNA and scrambled siRNA were purchased from Integrated DNA Technologies (Coralville, IA). Substrate for caspase-3, acetyl-Asp-Glu-Val-Asp-AFC, was obtained from MP Biomedicals (Solon, OH). The Cell Death Detection ELISA Plus kit was purchased from Roche Diagnostics (Indianapolis, IN). Protein Carbonyls Colorimetric Assay kit was purchased from Cayman Chemical (Ann Arbor, MI). The rabbit polyclonal antibody for Fpn1 was purchased from Alpha Diagnostic (San Antonio, TX). Alexa Fluor 680 conjugated anti-mouse IgG and IR-dye 800 conjugated anti-rabbit IgG were purchased from Invitrogen (Carlsbad, CA) and Rockland Inc. (Gilbertsville, PA), respectively. All solutions were prepared fresh prior to each assay.

### 2.2. Cell Culture

N27 cells were grown in RPMI-1640 medium containing 10% fetal bovine serum, 2 mmol/L L-glutamine, 50 units of penicillin, and 50 *μ*g/mL streptomycin and maintained at 37°C in a humidified atmosphere containing 5% CO_2_, as described in our previous publication [17].

### 2.3. Transient Transfections and Treatment Paradigm

N27 cells were transfected with hepcidin specific siRNA (hepcidin siRNA) or scrambled siRNA (control siRNA) using the Amaxa Nucleofector kit, following the manufacturer's instructions. Briefly, 3 × 10^6^ cells were resuspended in 100 *μ*L of the Nucleofector solution, along with 1.5 *μ*g of hepcidin siRNA or control siRNA, and subsequently subjected to electroporation using the Nucleofector program number A23. After 72 hours of initial transfection, cells were harvested and hepcidin mRNA was analyzed using quantitative real-time RT-PCR to confirm the knockdown efficiency. To evaluate the effect of hepcidin knockdown on 6-OHDA induced neurotoxicity, both control siRNA and hepcidin siRNA transfected cells were plated for 48 hours and treated with or without 100 *μ*M 6-OHDA for 6 hours. Cells were collected at the end of each treatment for the following experiments.

### 2.4. Quantitative Real-Time RT-PCR

Total RNA was isolated and converted to cDNA using the Absolutely RNA Miniprep kit and High Capacity cDNA Archive kit, respectively. Real-time PCR was performed using a Brilliant SYBR Green QPCR Master Mix kit and the Mx3000P QPCR system, as described in our previous publication [18]. The 18s rRNA was used as an internal control for quantifying RNA with the primer set purchased from SABiosciences (Valencia, CA). The reaction mixture included 2 *μ*L of cDNA, 12.5 *μ*L of 2x master mix, and 0.2 *μ*mol/L of each primer. Cycling conditions contained initial denaturation at 95°C for 10 min, followed by 40 cycles of denaturation at 95°C for 15 s and annealing at 60°C for 10 min. Fluorescence was detected during the annealing/extension step of each cycle. Dissociation curves were run to verify the singularity of the PCR products. The data were analyzed using the comparative threshold cycle method as described in our previous publication [18].

### 2.5. Western Blot Assay for Fpn1

Cell lysates were prepared using a modified radio immunoprecipitation assay (RIPA) buffer as described previously [19]. Equal amounts of protein were loaded for each sample and separated on 12% SDS-PAGE gels. After separation, the proteins were transferred onto a nitrocellulose membrane and were incubated with the rabbit polyclonal antibody directed against Fpn1 (1 : 1000) and developed with IR-dye 800 anti-rabbit secondary antibody (1 : 5000). *β*-actin was used as the loading control. Membranes were visualized on an Odyssey Infrared Imaging system (LICOR, Lincoln, NE).

### 2.6. Calcein Quenching to Measure Intracellular Iron Levels

The intracellular iron levels were determined by a calcein fluorescence quenching method modified from a previous study [20]. Calcein-AM is a membrane permeable, nonfluorescent molecule that becomes fluorescent by intracellular esterases. It is quenched rapidly by Fe^2+^ or Fe^3+^ and is a good indicator of the labile iron pool [20], which is cellular free iron or cheatable iron. The degree of quenching gives an estimate of the amounts of cellular cheatable iron. After the treatment, cells were incubated with calcein-AM in HEPES-buffered saline (HBS) for 30 min at 37°C. The excess calcein on the cell surface was washed off three times with HBS, and fluorescence was recorded using a Synergy II microplate reader (BioTek Instruments, Winooski, VT) at 485 nm excitation and 530 nm emission. Change in fluorescence intensity (with and without treatment after normalizing to protein concentrations) reflected the intracellular iron levels. Calcein fluorescence pictures were obtained with FLoid® Cell Imaging Station (Life Technologies).

### 2.7. Cell Viability Assays

Cell viability was measured using MTT assay as described earlier [17]. After each treatment, cells were incubated with serum-free RPMI medium containing 0.25 mg/mL MTT solution for 3 h at 37°C, followed by adding isopropanol-HCl (200 *μ*L) solution to dissolve intracellular purple formazan. The absorbance was read at 570 nm with a reference wavelength of 630 nm using a microplate reader (Molecular Devices, Sunnyvale, CA).

### 2.8. Caspase-3 Activity Assays

Caspase-3 activity was measured as described previously [21]. After treatment, the cell pellet after centrifugation was lysed with Tris buffer (50 mol/L Tris-HCl, 1 mmol/L EDTA, and 10 mmol/L EGTA at pH = 7.4) containing 10 *μ*mol/L digitonin for 20 min at 37°C. Lysates were subjected to quick centrifugation at 14,000 ×g and then incubated with a specific fluorescent substrate (Ac-DEVD-AFC, 50 *μ*mol/L) for 1 h at 37°C. The caspase-3 activity was measured with excitation at 400 nm and emission at 505 nm using a fluorescence microplate reader. The caspase-3 activity was expressed as fluorescent units/mg protein.

### 2.9. DNA Fragmentation Assays

DNA fragmentation assays were performed using the Cell Death Detection ELISA Plus kit as described previously [19]. After treatment, cell pellet was incubated with lysis buffer provided in the kit. The lysates were then centrifuged and the supernatant was incubated for 2 h with the mixture of HRP-conjugated antibody cocktail that recognizes histones and single- and double-stranded DNA. After washing away the unbound components, measurements were made at 490 nm and 405 nm using a fluorescence microplate reader. DNA fragmentation was expressed as absorbance units/mg protein.

### 2.10. Protein Carbonyl Assays

The oxidative damage to proteins was determined by measuring the protein carbonyl residues using DNPH (2,4-dinitrophenylhydrazine) according to the manufacturer's protocol. DNPH reacts with protein carbonyls to produce the corresponding hydrazones, which was measured spectrophotometrically at the wavelength of 360 nm. The carbonyl content was determined from the differences in absorbance between DNPH-reacted samples and nonreacted HCl samples and then standardized against the protein concentrations in the samples.

### 2.11. Statistical Analysis

Data were analyzed using the GraphPad Prism 5.0 (GraphPad Software, Inc., La Jolla, CA). All values were expressed as mean ± SEM. Student's *t*-test was used to compare the differences between groups. The values for two (control and hepcidin) siRNA transfected cells with 6-OHDA treatments were normalized to their respective controls (without 6-OHDA treatment) before statistical analysis. All the mean differences were considered significant at *P* ≤ 0.05.

## 3. Results

### 3.1. Downregulation of Hepcidin

**Figure 1:**
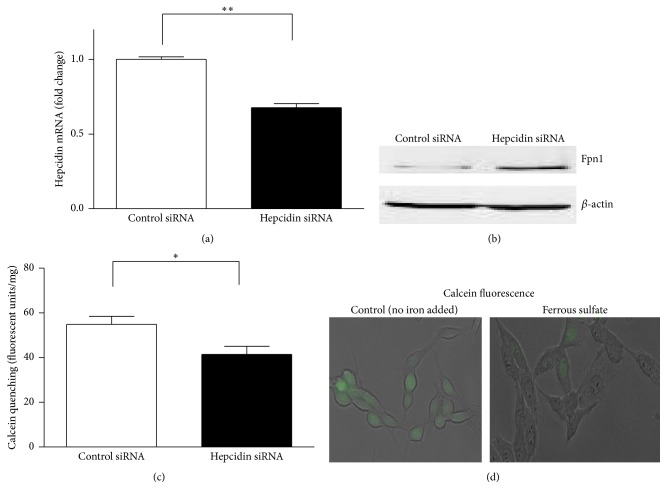
Effect of hepcidin knockdown on hepcidin mRNA levels measured by quantitative real-time RT-PCR ((a), *n* = 7-8), ferroportin 1 protein levels (normalized to *β*-actin) measured by Western blot (b), and intracellular iron measured by a calcein quenching method ((c), *n* = 6) in N27 cells. Representative calcein fluorescence images with and without incubation of 1 mM ferrous sulfate for 30 min are shown (d). Values are mean ± SEM. Differences between two groups were based on Student's *t*-test; ^*∗*^ *P* < 0.05; ^*∗∗*^ *P* < 0.0001. Control siRNA: scrambled small interfering RNA; hepcidin siRNA: hepcidin small interfering RNA; Fpn1: ferroportin 1.

To address the role of hepcidin in regulation of 6-OHDA induced neurotoxicity, we first utilized RNA interference (RNAi) technique to downregulate hepcidin levels in N27 dopaminergic cells. As shown in Figure 1(a), hepcidin mRNA levels were significantly downregulated (32.3%,  *P* < 0.0001) in hepcidin siRNA transfected cells compared with control siRNA transfected cells. Since hepcidin regulates Fpn1 by triggering its degradation, we further determined whether downregulation of hepcidin leads to increased Fpn1 protein expression (Figure 1(b)). Compared to the control siRNA transfected cells, the Fpn1 protein levels were elevated in hepcidin siRNA transfected cells, which confirmed a negative relationship between hepcidin and Fpn1 expression in dopaminergic cells. We also measured intracellular iron, which was indirectly measured by calcein fluorescence quenching, to ascertain whether decreased expression of hepcidin and increased expression of Fpn1 reduced intracellular iron levels. Calcein is the most widely adopted iron fluorescent probe that has been used to monitor changes of iron levels in a range of different cell types such as hepatocytes and colon cells [22]. As shown in Figure 1(c), hepcidin knockdown significantly decreased intracellular iron by 25% (*P* < 0.05). To confirm calcein fluorescence quenching by intracellular iron, we incubated the cells with or without 1 mM exogenous iron (ferrous sulfate in ascorbic acid solution, 1 : 44 molar ratio, pH 6.0) for 30 min and then examined the calcein quenching by fluorescence microscopy. As shown in Figure 1(d), supplementation of 1 mM ferrous sulfate effectively decreased calcein fluorescence.

### 3.2. Hepcidin Knockdown Protects N27 Cells from 6-OHDA Induced Cytotoxicity

**Figure 2:**
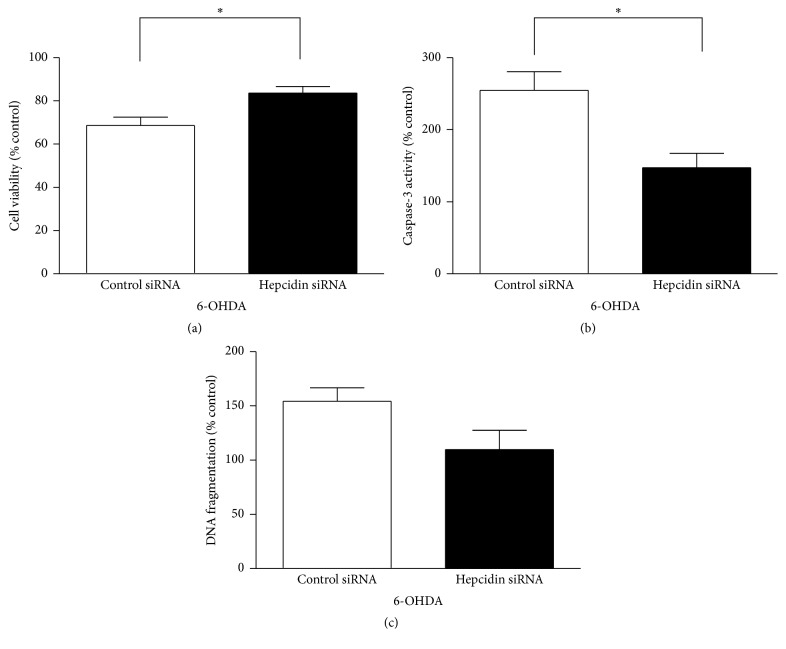
The role of hepcidin knockdown in 6-OHDA induced cytotoxicity measured by MTT ((a), *n* = 6), caspase-3 activity ((b), *n* = 4-5), and DNA fragmentation ((c), *n* = 4) in N27 cells. Cells were treated with 100 *μ*M 6-OHDA for 6 hours and the values (mean ± SEM) are normalized to their respective controls without 6-OHDA treatment; ^*∗*^ *P* < 0.05; difference between two groups was based on Student's *t*-test; control siRNA: scrambled small interfering RNA; hepcidin siRNA: hepcidin small interfering RNA.

All the values in Figure 2 were presented as percentage of respective controls without 6-OHDA treatment. We evaluated the effect of hepcidin knockdown on 6-OHDA mediated cell death using MTT assay (Figure 2(a)). Hepcidin knockdown moderately but significantly lessened the toxic effect of 6-OHDA by increasing cell viability by 22% (*P* < 0.05). When apoptosis was measured, hepcidin knockdown reduced 6-OHDA induced caspase-3 activity significantly (Figure 2(b), 42%, *P* < 0.05). DNA fragmentation was also reduced, but it was only marginally significant (Figure 2(c), *P* = 0.086). Together, these results demonstrate that hepcidin knockdown protects against 6-OHDA induced cell apoptosis.

### 3.3. Hepcidin Downregulation Reduces 6-OHDA Induced Protein Oxidative Damage and Intracellular Iron

As shown in Table 1, hepcidin knockdown decreased 6-OHDA induced protein carbonyls by 52% (*P* < 0.05) and intracellular iron by 28% (*P* < 0.01). These results show that hepcidin knockdown might protect against 6-OHDA induced neurotoxicity through attenuating oxidative stress by mediating intracellular free iron.

## 4. Discussion

Iron is an essential nutrient and is involved in many functions, such as acting as a cofactor for key enzymes involved in neurotransmitter biosynthesis [23]. On the other hand, excess free iron can cause significant oxidative stress by involvement in the production of hydroxyl radical formation, glutathione consumption, protein aggregation, lipid peroxidation, and nucleic acid modification [24, 25]. The body iron homeostasis is regulated by iron regulatory proteins to minimize the amount of free iron available to participate in free radical formation. Among those proteins, hepcidin is considered as a principal regulator because of its function to inhibit cellular efflux of iron by binding to Fpn1 at the cell surface and inducing its subsequent degradation [26].

Recent studies have suggested a critical role for hepcidin in a variety of disorders, including anemia of inflammation, chronic kidney disease, and familial hemochromatosis [26–28]. However, the participation of hepcidin in neurodegenerative disorders is very limited. In our earlier study on cell culture [29], 6-OHDA increased the expression of hepcidin and decreased the expression of Fpn1, which made us design this current study to investigate the effect of hepcidin with knockdown experiments. We used N27 dopaminergic neuronal cell model to detect hepcidin and Fpn1 expression, since N27 cell line possesses all physiological and biochemical properties of dopaminergic neurons [30]. Our results show that both hepcidin and Fpn1 are expressed in N27 cells and that knockdown of hepcidin remarkably increased Fpn1 expression and reduced intracellular iron levels as measured by calcein quenching. These results are consistent with previous studies, which demonstrate that hepcidin is widely expressed in murine brain and might play a key role in regulating iron levels in the brain by downregulating Fpn1 expression [15, 31, 32].

Our study also shows that regulation of brain iron efflux by hepcidin may play a protective role in 6-OHDA induced neurotoxicity. Hepcidin knockdown and subsequent upregulation of Fpn1 protein significantly attenuated the protein oxidative damage induced by 6-OHDA, ultimately leading to a reduction in cell apoptosis, as evidenced by decreased caspase-3 activation and marginally decreasing DNA fragmentation. Increasing caspase-3 activity and DNA fragmentation, respectively, more than 2- and 1.5-fold in the control siRNA cells but only showing a small increase in hepcidin siRNA cells with 6-OHDA treatment clearly shows the protection with hepcidin downregulation, even with low knockdown efficiency. 6-OHDA is a hydroxylated analogue of the neurotransmitter dopamine and represents a classic neurotoxin used for the initiation of the PD neurodegeneration both in vitro and in vivo [33]. Studies have shown abnormal iron accumulation in 6-OHDA induced PD models, and 6-OHDA induced neurotoxicity may result from free iron and the ensuing production of free radical species [34]. However, the precise mechanism underlying abnormal iron accumulation in 6-OHDA induced neurotoxicity is not very clear. Song et al. [35] demonstrated that upregulation of iron regulatory protein 1 (IRP1) might be responsible for decreased expression of Fpn1 and increased cellular iron accumulation. Another study reported that divalent metal transporter 1 (DMT1) + IRE upregulation is involved in 6-OHDA induced iron accumulation and aggravated oxidative injury [36]. Our study provides direct evidence for the first time that hepcidin-ferroportin axis at least partially accounts for iron accumulation in 6-OHDA induced neurodegeneration. Hepcidin knockdown resulted in upregulation of Fpn1, which may enhance iron release and alleviate iron accumulation in dopaminergic neurons, and eventually protected neurons from 6-OHDA induced apoptosis. Our data with calcein quenching support this relationship. In addition to its role in iron homeostasis, hepcidin is also recognized as a principal mediator in inflammation [37, 38], which is also directly linked to the pathogenesis of PD [39, 40]. Thus, further study is needed to study the linkage between hepcidin expression, iron status, and neuroinflammation in PD. In addition, research has shown that 6-OHDA can induce oxidative damage and neurotoxicity of both the peripheral and the central nervous system [41], and hepcidin is strongly expressed in other brain regions such as the olfactory bulb [42]. Future research is needed to investigate whether hepcidin has a role in protecting from 6-OHDA induced neurotoxicity in other brain regions.

## 5. Conclusions

In conclusion, our study demonstrates that hepcidin plays an important role in iron accumulation, thus causing oxidative stress and associated neurotoxicity. Hence, the approaches that can reduce hepcidin and increase Fpn1 expression might be effective strategies in preventing the progression of PD.

## Figures and Tables

**Table 1:** The role of hepcidin knockdown in 6-OHDA induced oxidative damage measured by protein carbonyls (*n* = 4) and intracellular iron measured by calcein quenching method (*n* = 6).

	Protein carbonyls (nmol/mg protein)	Calcein quenching (fluorescent units/mg protein)
Control siRNA	18.5 ± 2.9	112.4 ± 4.8
Hepcidin siRNA	8.9 ± 1.1^*∗*^	80.8 ± 6.2^*∗∗*^

Values are mean ± SEM; ^*∗*^ *P* < 0.05; ^*∗∗*^ *P* < 0.01. Differences between two groups were based on Student's *t*-test; control siRNA: scrambled small interfering RNA; hepcidin siRNA: hepcidin small interfering RNA.

## Abbreviations

PD: Parkinson's disease
6-OHDA: 6-Hydroxydopamine
SN: Substantia nigra
MPTP: 1-Methyl-4-phenyl-1,2,3,6-tetrahydropyridine
Fpn1: Ferroportin 1
RIPA: Radio immunoprecipitation assay
HBS: HEPES-buffered saline
DNPH: 2,4-Dinitrophenylhydrazine
RNAi: RNA interference
IRP1: Iron regulatory protein 1
DMT1: Divalent metal transporter 1
IRE: Iron responsive element.
